# Stress-Controlled Torsional Fatigue Assessment of Selected Contemporary Endodontic Instruments

**DOI:** 10.1007/s00784-026-06811-0

**Published:** 2026-03-14

**Authors:** Kushagra Ohri, Philip Yuan-Ho Chien, Ove A. Peters

**Affiliations:** 1 School of Dentistry, The University of Queensland, Brisbane, Australia; 2 Oral Health Centre, School of Dentistry, The University of Queensland, 288 Herston Road, Herston, Qld 4006 Australia

**Keywords:** Nickel-titanium, Stress-controlled torsional fatigue, Ultimate torsional strength, Endodontic instruments

## Abstract

**Objectives:**

To compare stress-controlled torsional fatigue resistance of TruNatomy (TN), ProTaper Next (PTN), and ProTaper Ultimate (PTUL) instruments of comparable sizes at both room and body temperature.

**Materials and Methods:**

Torsional strength and related twist angles were determined at both temperatures in a torque testing fixture. Stress-controlled torsional fatigue resistance (TFR) was determined by cycling instruments in a custom fixture to pre-set twist angles corresponding to 90% torsional strength until fracture. Data were analyzed using two-way ANOVA and Tukey post-hoc tests with the significance level set at 0.05.

**Results:**

Overall, mean (± standard deviation) TFR ranged from 192 (± 66) to 841 (± 201) cycles; PTUL had the highest TFR and TN the lowest; the differences among the three instruments were statistically significant. At 37 °C compared to room temperature, PTUL had a similar TFR, while PTN had a higher and TN a lower one. Applying customized stress-controlled conditions resulted in differences in TFR among the 3 instrument types. Different environmental temperatures had varying effects on the tested instruments manufactured from differently heat-treated nickel-titanium (NiTi) alloy.

**Conclusions:**

Understanding TFR is relevant for clinical use of NiTi instruments for continuous rotation and reciprocation, specifically for motor settings. Within the limitations and specific testing parameters of this study, stress-controlled TFR varied among the three tested instruments. Different environmental temperatures had varying effects on the tested instruments manufactured from differently heat-treated nickel-titanium (NiTi) alloy. Variations in instrument design and the NiTi alloy type used may account for different torsional fatigue performance and suggest specific clinical usage parameters.

**Clinical Significance:**

During canal preparation, instruments are stressed under cyclic load, a condition which is replicated in this experiment. The data is relevant for motor presets for continuous rotation and reciprocating motion as well as handling of contemporary heat-treated Nickel-titanium instruments.

## Introduction

The use of nickel-titanium (NiTi) instruments is widespread globally, mainly due to their improved ability to prepare complex root canal systems [1]. Moreover, the motorization of NiTi instruments has played a role in reducing complications frequently associated with traditional stainless-steel files, such as the formation of ledges, zips, perforations and transportations within the root canal system [2]. However, NiTi instruments are not immune to fracture, and fractured instruments in the root canal can physically obstruct the ability to adequately perform cleaning and shaping. The biomechanics of NiTi instrument fractures have been recently reviewed with specific considerations of torsional fatigue resistance (TFR) [3].

In root canal preparation with engine-driven instruments and under the constraints of the specific canal anatomy, there is an interplay of torsional loading and fatigue with the process of crack nucleation [3]. Importantly, the two distinct types typically described for instrument fracture, namely torsional (ductile) failure and cyclic (brittle) fatigue failure [4], are not representative of clinical conditions [2].

In the engineering literature, three experimental modalities have been previously described for evaluating fatigue behavior of nickel-titanium: stress-controlled testing (stress-life), strain-controlled testing (strain-life) and damage-tolerant analysis [5, 6]. Stress-controlled experiments involve a cyclic test under fixed force amplitudes irrespective of the displacement, whilst strain-controlled tests are performed under fixed strain amplitudes, irrespective of the force required to achieve those displacements [7]. The damage-tolerant approach applies fracture mechanics to a distinct set of material properties and a pre-established crack size to provide a conservative prediction to fatigue life [8].

A strain-controlled design [9], based on twist angles and applied to a single instrument, may provide initial information for TFR. However, to compare different instrument designs a stress-controlled design [10, 11] is preferable because canal preparation is inherently a function of the stress developing in a instrument while cutting dentin [3]. Such as stress-controlled experiment would involve setting instrument- and location-specific torque thresholds in order to derive clinically applicable performance indicators.

Importantly, even recent studies testing TFR do not account for some important variables such as environmental temperature [12] and relevant differences in instrument design and alloys. Therefore, the present experiments aim to address this gap in knowledge by determining stress-controlled TFR of selected contemporary rotary instruments manufactured from different alloys tested at simulated body temperature. The null hypotheses were that there are no differences in TFR among the tested instruments and between temperatures.

## Materials and Methods

The experiments described here included a total of 180 rotary instruments with 60 each of the following: TruNatomy (TN) Prime (tip size 0.26 mm), ProTaper Next (PTN) X2 (tip size 0.25 mm) and ProTaper Ultimate (PTUL) F2 (tip size 0.25 mm) (Dentsply Sirona, Charlotte, NC, USA).

Sample size estimates for the two experiments described below were based on previously published data for UTS [4] and for strain-controlled TFR [9], respectively. Specifically, the expected effect sizes (Cohen’s D) for UTS tests was estimated to be about 4.5 whilst in the absence of comparable stress-controlled data, strain-controlled comparisons suggested an effect size of about 3.5.

Twelve instruments from each of the 3 groups were randomly selected to determine the ultimate torsional strength (UTS) at 6 mm from the tip (D6). Six instruments per group were tested at 22 °C and 6 were tested at 37 °C (Table 1). Determination of UTS was broadly performed according to ISO 3630-1:2019 standards, with the exception that the level of fracture from the tip was at 6 instead of 3 mm. The torque testing device [4, 13] has been described in detail elsewhere; in brief, it consisted of a torque sensor and a motor, mounted on a metal base. A instrument was clamped in a soft brass chuck and rotated at 2 revolutions per minute (rpm); torque was continuously recorded (in Newton-centimeters, Ncm) over time (in milliseconds, ms), whilst twist angles were calculated off-line based on the rpm setting.

**Table 1:** Allocation of samples for each instrument, condition and test (see text for details)

		UTS	TFR	Total
Room Temperature	TruNatomy Prime	6	24	30
	ProTaper Next X2	6	24	30
	ProTaper Ultimate F2	6	24	30
Body Temperature	TruNatomy Prime	6	24	30
	ProTaper Next X2	6	24	30
	ProTaper Ultimate F2	6	24	30
				180

For the instruments tested at simulated clinical conditions, an ambient temperature of 37 °C (± 2 °C) was maintained with a fan heater. At the start and end of every test cycle, temperature at the surface of the instrument was confirmed by using a Ti9 infra-red thermal imager (Fluke Corp., Everett, WA, USA) with an accuracy of 5% and a temperature range of -20 °C to + 250 °C. Surface temperature of the instruments at room temperature measured approximately 22 °C. Since the emissivity of the Ti9 imager was fixed at 0.95 (95%), the shanks of the instruments were painted black to render the surface efficient in radiating energy. Data obtained from the torque testing platform for UTS and time to instrument fracture was recorded for off-line analysis. Subsequently, values for 90% of maximum torque before fracture (90% UTS) and the time taken to reach this value were calculated for each instrument. These values were then used to derive mean twist angles required by instruments from each group to reach 90% UTS at room temperature and at 37 °C.

Mean twist angles served as pre-sets of cyclic loading for a customized stress-controlled test to determine TFR. Each cycle was defined as starting in a clockwise direction from a start position of zero torque in the instrument to the pre-set angle and then back to start position in an anticlockwise direction. Therefore, the maximum amplitude in the cycle was when an instrument was subjected to a torque value equivalent of 90% UTS and the minimum amplitude was zero torque, both of which occurred at D6. Each instrument was tested for TFR at the manufacturer’s recommended speed of rotation (PTN-300 rpm, TN-500pm, PTUL-400 rpm).

**Fig. 1:**
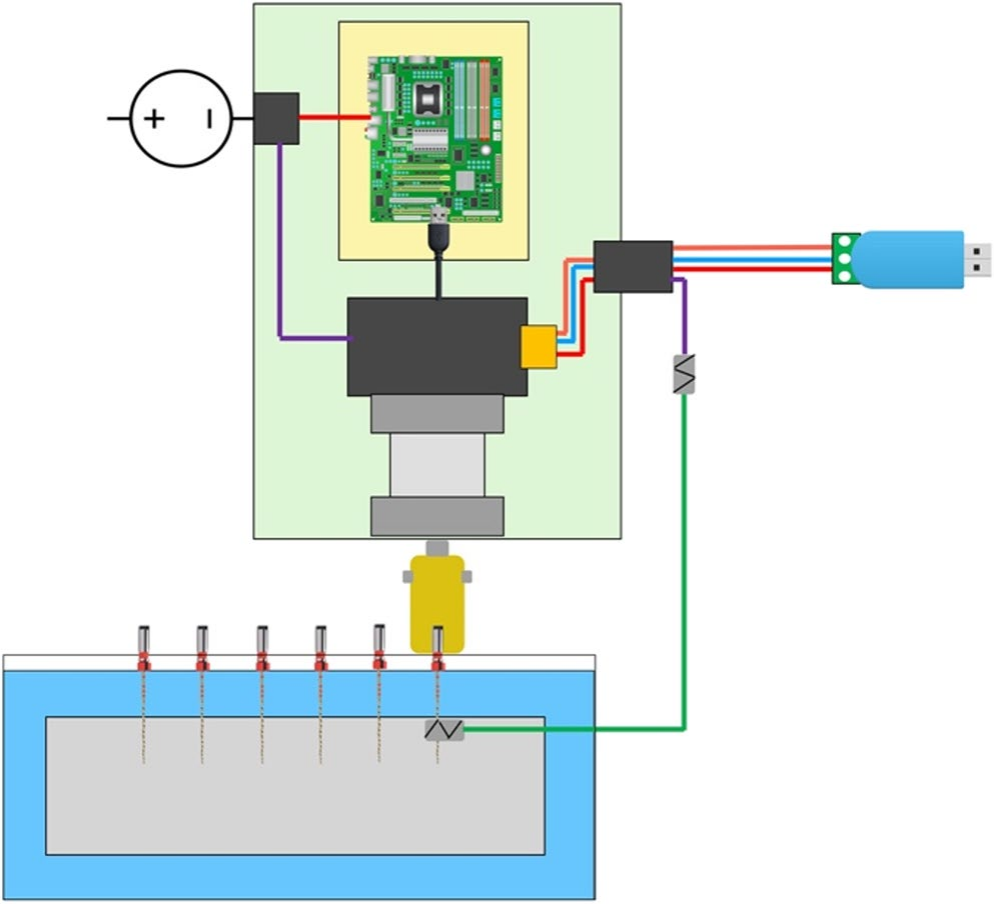
Schematic diagram of motor assembly and testing set-up. Instruments were placed in the fixture as described and tested in random sequences in a water bath (blue shade) at room or body temperature, respectively. A lead connected the embedded instrument portion to the controller; its interruption stopped the cycle counter and motor after instrument fracture

The experimental setup for TFR test comprised of a custom designed motor assembly to which the shank of the instrument was attached using a specially made coupling (Fig. 1). The mounting assembly was made up of galvanized steel bracket plates with openings that accepted 6 instruments per run. These were placed such that the tip of a instrument protruded 2 mm through the perforated plate and 4 mm of shank was surrounded by a perforation in the plate. Flowable composite resin (Filtek Supreme, 3 M ESPE, North Ryde, NSW, Australia) was used to secure each instrument in a perforation of the bracket plate.

Mounting was performed under 4.3X magnification using EyeMag loupes (Carl Zeiss, Oberkochen, Germany). To confirm the instrument mounting accuracy, photographs of mounted instruments were taken with a digital single-lens reflex camera Canon EOS100 D (Canon, Tokyo, Japan) and a macro-lens (Canon MP-E65mm f/2.8 1-5X Macro) and ring-light flash (Canon MR14-EX II) prior to being assessed using ImageJ (Public Domain license, U.S. National Institutes of Health, Bethesda, MD, USA) at 300x digital zoom. Mounting accuracy at D6 was ± 0.28 mm.

A stepper motor (17MDSI202S Anaheim Automation, Anaheim, CA, USA) with an accuracy of 0.9° in rotation was used for cycling the instruments. The motor was mounted in a waterproof case (Jaycar, Rydalmere, NSW, Australia) and connected to a USB RS-485 dongle (Core Electronics, Newcastle, NSW, Australia), which in turn connected the computer output to the motor (see Fig. 1). A total of 24 instruments were tested at each of the two temperatures and for each instrument type (Table 1).

Custom software was written in LabVIEW (National Instruments, Austin, TX, USA) for both controlling motor function, including stopping cycles at instrument fracture, and for counting cycles to fracture as a measure of TFR. Specifically, when the fatigue cycling started, the software was set to count the number of cycles in a way that a half cycle was when the instrument rotated clockwise to 90% UTS and another half was when it rotated back to the starting position. As a result of instrument fracture, a break in continuity occurred between the lead connecting the instrument holder with the remainder of the circuit, which then automatically stopped the motion of the motor. At this point, the program paused the cycle count and the number of cycles to fracture was recorded.

Data are presented as means and standard deviations (SD) for UTS and twist angle at fracture for stationary loading as well as number of cycles to fracture to as a measure of TFR. Comparisons were made between room temperature and body temperature among instruments.

The Shapiro-Wilk test was used to examine the data and confirmed normality for all data and variances were considered sufficiently homogenous based on Bartlett’s test. Subsequently, statistical analyses (Prism Version 9.5.1, Graph Pad, Boston, MA, USA) were performed with two-way analysis of variance (ANOVA) with Tukey’s post-hoc tests. The significance level was set at 0.05.

## Results

### Stationary Loading (UTS and Twist Angle)

**Fig. 2:**
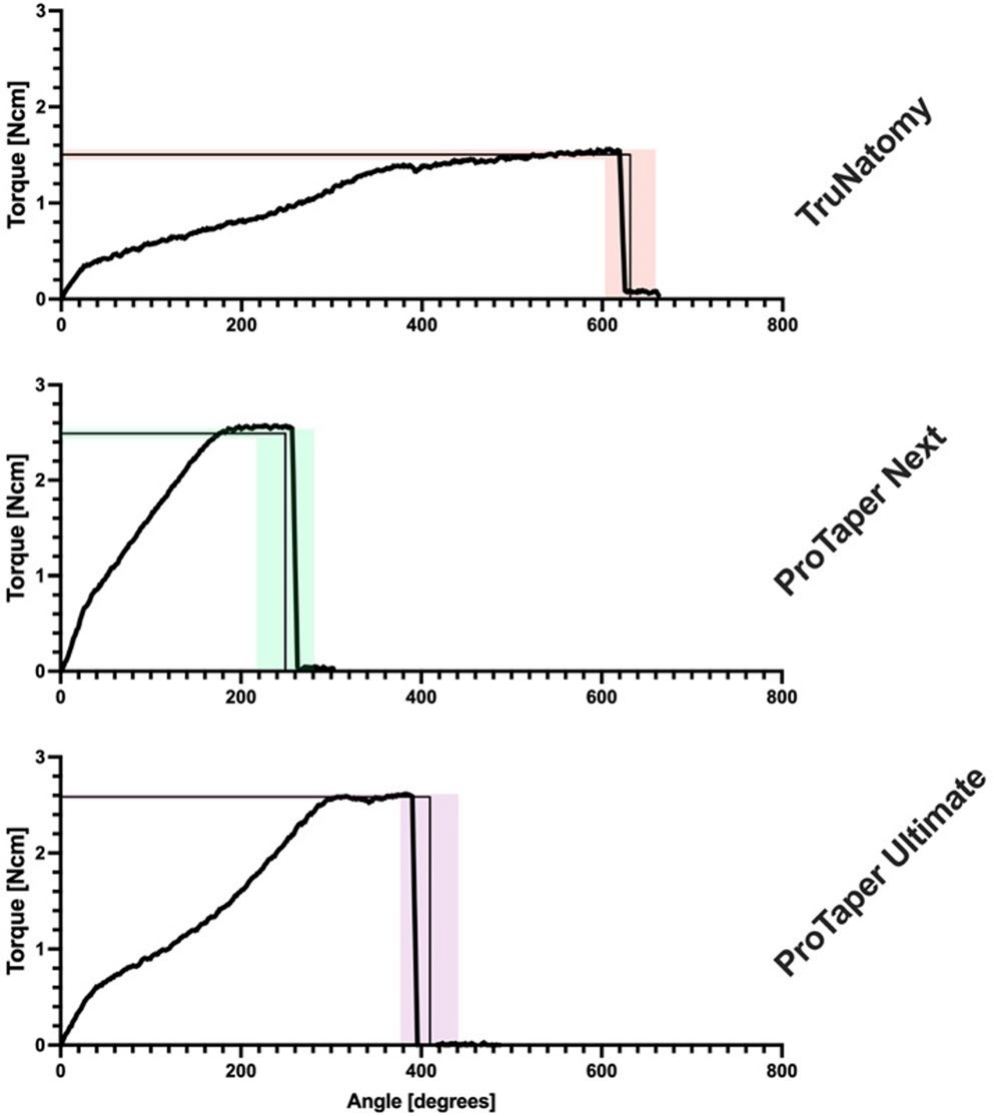
Data for representative examples of stationary fracture for the three instruments tested at body temperature (lines indicate means for ultimate torsional strength and twist angle at fracture, while shaded areas represent ± SD, *n* = 6). Note torque to fracture was determined at D6

The data from stationary loading at D6 is presented in Table 2 as well as in Fig. 2. ANOVA confirmed overall significant differences for UTS (F = 254.72; 2 DF; *p* < 0.0001) and twist angle (F = 131.43; 2 DF; *p* < 0.0001) at D6 among instruments, whilst there was no significant effect of temperature (F = 0.36; 1 DF; *P* = 0.553).

**Table 2:** Stationary fracture data for instruments tested at room and body temperature (*n* = 6 per group). Note fracture level was at D6. Both Ultimate Torsional Strength (UTS) and twist angles were significantly different among instruments (see text for details)

		UTS range [Ncm]	Twist angle [degrees]	Mean UTS [Ncm] (± SD)	Mean twist angle [degrees] (± SD)
*Room temperature*	TruNatomy Prime	1.32–1.63	574–660	1.52 (0.10)	629 (33)
	ProTaper Next X2	2.14–2.58	246–275	2.40 (0.17)	255 (11)
	ProTaper Ultimate F2	2.60–2.82	376–637	2.66 (0.13)	505 (90)
*Body temperature*	TruNatomy Prime	1.37–1.74	542–675	1.49 (0.14)	631 (54)
	ProTaper Next X2	2.30 - 2.67	192–322	2.44 (0.13)	249 (61)
	ProTaper Ultimate F2	2.47–2.68	326–482	2.57 (0.08)	412 (61)

Post-hoc tests demonstrated that both at room and body temperature, UTS was ranked as: PTUL > PTN> TN (Table 2), with all pairwise comparisons showing significant differences (*P* < 0.01). Similarly, at both room temperature and body temperature, the twist angle to fracture was ranked as TN> PTUL > PTN (Table 2). Also in this case, all pairwise comparisions between instruments showed significant differences (*P* < 0.001).

At 90% UTS torque values for TN were in the extended plateau of the deformation phase for some instruments (Fig. 2) whereas for PTUL and PTN that level was always prior to the plateau phase. The presets for the cyclic loading test (90% UTS and corresponding twist angles) are listed in Table 3.

**Table 3:** Ultimate torsional strength (UTS) data used to calculate presets for torsional fatigue resistance (TRF) tests

		90% Max. Torque [range, Ncm]	Time to 90% UTS [range, ms]	Set twist angle [degrees]	Predicted torque [Ncm]
Room temperature	TruNatomy Prime	1.19–1.41	30,200 − 36,700	403	1.37
	ProTaper Next X2	1.93–2.32	12,500 − 13,700	155	2.15
	ProTaper Ultimate F2	2.22–2.54	22,200 − 25,700	291	2.40
Body temperature	TruNatomy Prime	1.23–1.56	27,300 − 33,700	373	1.34
	ProTaper Next X2	2.07–2.40	10,100 − 13,900	142	2.20
	ProTaper Ultimate F2	2.23–2.41	21,900 − 22,600	268	2.31

### Cyclic Loading (Torsional Fatigue Resistance)

**Fig. 3:**
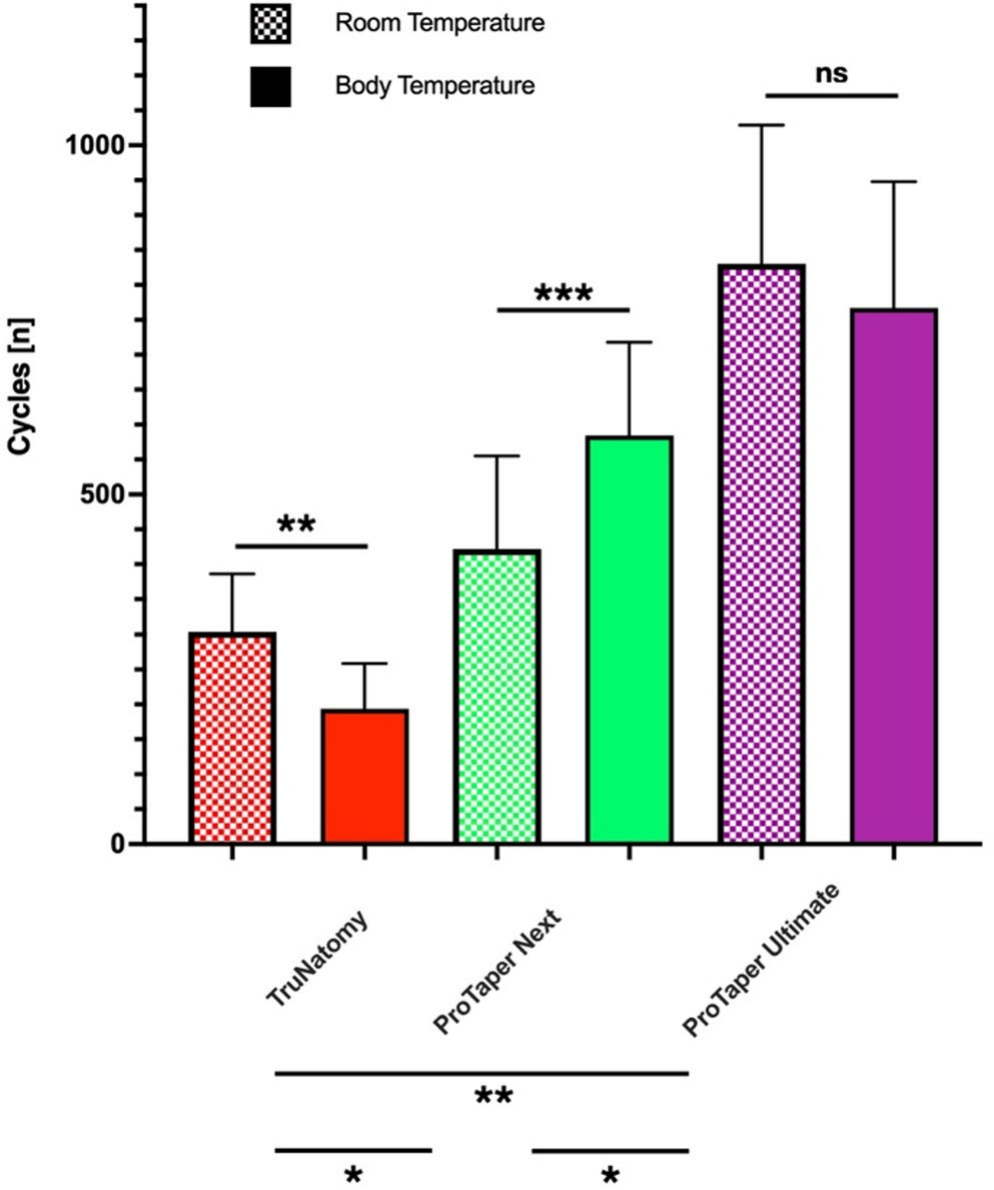
Torsional resistance in stress-controlled conditions, expressed as numbers of cycles (±SD, *n* = 24) to fracture at room and body temperature. Significant differences using ANOVA and Tukey’s post-hoc tests are indicated by asterisks, ns (not significant)

The data for mean number of cycles to fracture in torsion are presented in Fig. 3. ANOVA confirmed significant differences among instruments (F = 190.9; 2 DF; *P* < 0.001). The effect of temperature alone (F = 0.03; 1 DF; *P* = 0.869) was not significant but there were significant interactions (F = 13.57; 2 DF; *P* < 0.001) for each instrument; the findings are summarized as follows according to Tukey’s post hoc tests. At both room and body temperature, TFR was ranked as PTUL > PTN> TN and the difference between the three was overall statistically significant (*P* < 0.001).

Compared to room temperature, TFR for TN decreased at body temperature; the difference was statistically significant (*P* < 0.01). Conversely, compared to room temperature, the TFR for PTN increased at body temperature and the difference was statistically significant (*P*<0.0.001). Tested at room and body temperature, PTUL had statistically similar TFR. Collectively the null hypotheses were therefore partially rejected.

## Discussion

Torsional fatigue, as opposed to torsional loading, is a clinically relevant yet incompletely understood variable [3]. Earlier work mostly used a strain-controlled approach to determine torsional fatigue resistance [9, 12]. When comparing instruments with vastly different UTS, a stress-controlled approach is preferrable for TFR [3] and testing heat-treated NiTi instruments at body temperature is decisive for clinical transferability [14]. This study evaluated TFR of 3 types of contemporary NiTi instruments by cycling them at torque values of 90% of UTS. These constraints were selected in line with earlier studies [15] so that instruments experienced relatively high levels of torsional stress but were expected to retain their ability to cut dentin without fracture.

It is understood that the magnitude of torsional stress experienced by a rotating instrument within a canal is dependent on the preoperative canal volume [13], apical force applied by the operator, the wall contact area [16] and its cross-sectional design [17]. Collectively, these parameters suggest that real-world torsional behavior of a instrument may be better tested by a customized stress-controlled experiment, rather than in strain-controlled conditions. Thus, the experimental design subjected NiTi instruments to 90% of UTS and not to a specific amount of strain level, which is typical for flexural fatigue studies for NiTi instruments [15]. In addition, at 90% UTS, NiTi and other alloys are still likely to eventually fracture in fatigue because permanent slip within crystal grains form cracks with cycling even below the elastic limit of the material [18].

As mentioned, the primary outcome addressed in this study was to evaluate the number of cycles to fracture at room temperature and body temperature, as an indication of TFR of NiTi instruments. At room temperature, PTUL required a higher number of cycles to fracture compared to both PTN, whilst TN required the lowest number of cycles to fracture. It should be noted, however, that manufacturer presets for the three tested instruments varies as well, with TN used expressly at a low torque limit of 1.5Ncm as opposed to PTN (2-3Ncm) and PTUL (4Ncm). Moreover, while the tip sizes were comparable, in the present experiment fatigue fractures occurred at D6, with related differences in cross-sectional diameters. At that level, PTN and PTUL are similar in size (0.67 mm) while TN is smaller (0.57 mm) with resultant different torsional resistances. Selecting D6 rather than D3 for the fracture location ensured that UTS for a small flexible instrument such as TN Prime was in a range of the manufacturer recommended torque setting and was similar to a prior study [10].

Contemporary NiTi instruments tested here for UTS fractured in their plastic deformation phase. This phase was represented by the plateau region in the stationary fracture records (Fig. 2). This region presents a gradual overall increase in torque between the start of plastic deformation until the point where the instrument ultimately fractures. This is unlike torque, where a steep change occurs from the start of torsional resistance testing until a NiTi instrument reaches the plastic deformation phase. The noted different behavior of TN could be considered of clinical benefit since plastic deformation is noticeable and a instrument can be discarded.

On the other hand, the longer plastic deformation phase before fracture for TN meant that 90% UTS values were located just before or in the plastic deformation phase for this instrument. Despite gradual increase in torque values in this phase, a longer plateau meant TN had greater than 10% variability in torque values in this phase. Consequently, the pre-set twist angle derived from the 90% UTS value may have placed the maximum cycling amplitude for TN close to or in its plastic deformation phase. In contrast, PTN and PTUL had an obviously shorter plastic deformation phase prior to fracture. In fact, the 90% UTS values (and the pre-set angles) for these 2 instrument groups were below torque levels required to cause plastic deformation. Indeed, one may speculate that PTUL and PTN are better suited than TN for reciprocating motion.

TN fractured at the lowest number of cycles at both room and body temperature when repeatedly stressed to 90% UTS. It is well established that at such high-stress amplitudes, metal undergoes low cycle fatigue as a result of localized cyclic plastic strain and has comparatively short fatigue lives [19]. In addition, reversibility of phase transformation in NiTi alloy is found to be severely affected by plastic strains and crack growth within the slip bands is encouraged by plasticity [18]. Another factor that could play a role in fatigue behavior of TN is the higher rotational speed as strain rate in fatigue testing increases the rate of accumulation of dislocation densities in austenitic alloys and reduces critical stress for inducing martensite phase [20]. There is a significant increase in probability of endodontic instrument distortion and fracture for austenitic alloy by increasing rotational speed [21]. However, the impact it had in this experiment when compared to the role played by inherent material properties of mixed phase instruments is not quantifiable and probably negligible in size.

The results indicate PTUL has better TFR compared to PTN. Both instruments differ in their surface, taper, pitch, diameter, and cross section and these details may play a role in their fatigue behavior; it is difficult to quantify the individual impact of each variable [22]. However, NiTi fatigue resistance is influenced mostly by thermomechanical treatment [23]. Specifically, the physical properties of NiTi are impacted by its phases and any property of the alloy acting as a proxy for a volume fraction of martensite [24]. As such, the differences in TFR between PTN and PTUL could be explained from the current knowledge of metallurgy and post-manufacture heat treatment of these instruments.

The three instruments tested were manufactured from different materials, with PTN consisting of pre-manufacture heat-treated M-wire, while PTUL and TN showed specific colors indicating variations in post-manufacture heat treatment [25–27]. As a result of their respective heat treatments, these instruments comprise of mixed phase alloys where PTN is predominantly austenitic and PTUL is in the martensitic phase at room temperature [28].

Only a few prior studies examined TFR of NiTi instruments and collectively suggested that NiTi instruments with austenitic alloy performed better than those with predominantly martensitic alloy [10, 11, 29, 30] and the data presented here are in apparent contradiction those studies. This apparent variance may be explained by the methodological design of previous studies, which subjected all instruments to the same amount of torsional stress. Under such experimental conditions, stiffer austenitic instruments are expected to perform better than those in martensitic and mixed phase conditions. This is because the displacement of the austenitic alloy specimen (in this case NiTi instruments) per cycle is less, as is the total energy per cycle [5, 31].

This study also presents with some limitations; for example, under clinical conditions torsional stresses experienced by the NiTi instrument are not always localized at D6 and fatigue properties may vary at different points on the instrument. Prior experiments selected different fracture points, e.g., D3 [11] or D5 [10, 29], non-clinical rotational speeds, e.g., 50rpm [29] and global torque settings such as 0.5Ncm [11] or 1Ncm [10, 29], essentially precluding any direct comparisons.

The present study was also limited to instruments designed for continuous rotation; understanding stress-controlled TFR is relevant for recommended torque settings for those instruments. However, it may be even more important to investigate contemporary reciprocating instruments [12] with different designs and under simulated clinical conditions to specify individual presets for flexible reciprocating instruments.

Testing NiTi instruments to UTS at 2 rpm and for cyclic fatigue resistance without torsional load is suitable for production consistency (e.g., as described in ISO3630-1) but these tests do not represent cyclic loading conditions present in clinical use [2, 15]. The present experimental setup considers the inherent metallurgical differences between the instruments used under clinically relevant conditions such as body temperature, customized stress levels and manufacturer recommended rpm. However, individual clinical usage, such as hand movements and reprocessing methods, was modelled in the present experimental design.

In conclusion, and within the limitations and specific testing parameters of the present study, PTUL performed best in TFR both at room and body temperature, PTN performed better than TN but worse than PTUL, at both body and room temperatures. As a result of increase in temperature, fatigue resistance in torsion was reduced for PTUL and TN but it increased for PTN. Overall, variations in instrument design and differences in nickel-titanium alloy used resulted in variations in stress-controlled TFR. Consequently, the reported data is relevant for clinical usage parameters and motor settings.

## Data Availability

Data is available from the corresponding author upon reasonable request.
